# Assessment of the Distribution and Safety of *Tetragenococcus muriaticus* for Potential Application in the Preparation of Chinese Grasshopper Sub Shrimp Paste

**DOI:** 10.3389/fmicb.2021.628838

**Published:** 2021-01-28

**Authors:** Xue Sang, Xinxiu Ma, Yanan Zhang, Hongshun Hao, Jingran Bi, Gongliang Zhang, Hongman Hou

**Affiliations:** ^1^School of Food Science and Technology, Dalian Polytechnic University, Dalian, China; ^2^Liaoning Key Lab for Aquatic Processing Quality and Safety, Dalian, China

**Keywords:** *Tetragenococcus muriaticus*, grasshopper sub-shrimp paste, safety assessment, flavor improvement, histamine control

## Abstract

The bacterial profiles of 63 grasshopper sub shrimp paste samples collected from seven typical regions around the Bohai Sea were investigated by high-throughput sequencing. *Tetragenococcus muriaticus* was found to be the prevailing species present in all the samples, and the presence of *T. muriaticus* also weakly correlated with the histamine content in the samples. Six *T. muriaticus* strains with low biogenic amine (BA)-producing ability and deficient in histamine production were identified and subjected to safety assessment. All six strains displayed weak resistance to fifteen known antibiotics as based on the *Enterococcus* breakpoint values. None of the strains exhibited hemolytic activity or biofilm formation. All strains exhibited were able to grow on MRS agar containing 21% NaCl and expressed amine oxidase and strain-specific proteases and lipases. Most of the strains exhibited acid production at 18% NaCl. Moreover, three of the strains (designated as SG, TS, and QH) with histamine degradation ability were inoculated into separate shrimp paste samples to determine their effect on BA accumulation. The results indicated that the addition of *T. muriaticus* to shrimp pastes not only led to a significant reduction of BA content in the pastes but also improved the flavor of the pastes. Consequently, these strains may be used as potential candidates for controlling the content of histamine in fermented foods.

## Introduction

**FIGURE 1:**
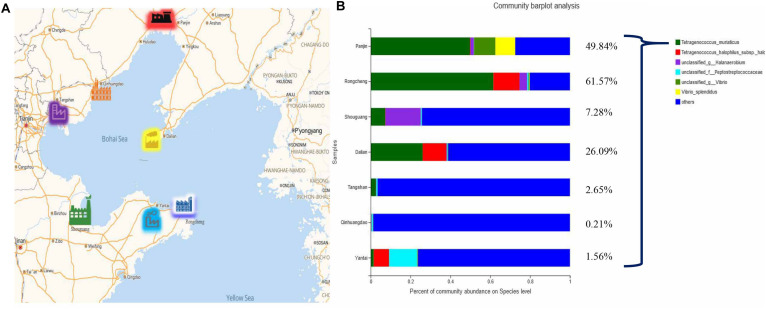
Regional distribution of grasshopper sub shrimp paste samples and community composition of bacteria at the species level. **(A)** Regional distribution of grasshopper sub shrimp paste samples; **(B)** Community composition of bacteria at the species level (Others merger <0.07).

Grasshopper sub shrimp paste is a famous traditional fermented aquatic product in China. Grasshopper sub shrimps grow in water bordering the junction of seawater and freshwater. Bohai Sea is an inner sea surrounded by the “C” shape of Liaoning Peninsula, Shandong Peninsula and North China Plain, covering more than half of China (Figure 1A), and a large amount of organic substances found along the coast of Bohai Sea makes it a rich habitat for shrimps. Therefore, grasshopper sub shrimp paste factories in China are located around the Bohai Sea (Sang et al., 2020a). Due to its unique flavor and excellent nutritional value (Lv et al., 2020; Sang et al., 2020b), grasshopper sub shrimp paste is popular in China. However, the process of natural fermentation involved in the typical production of grasshopper sub shrimp paste has made the process susceptible to contamination by microbes from the environment, therefore, making the process less hygienic. No control measures are implemented in the fermentation process, and this can sometimes result in a lack of quality and safety control, thereby affecting both the quality and safety of the fermented food, such as shrimp paste.

The production of shrimp paste is a complex process whereby the endogenous proteases in the shrimps and those produced by halophilic bacteria surviving under high salt condition induce the formation of low molecular weight compounds (such as peptides, amino acids, aldehydes, organic acids and amines), which can then affect the safety and flavor of the fermented products (Pongsetkul et al., 2017; Lv et al., 2020). Biogenic amines (BAs) can easily accumulate in protein-rich fermented foods, with excessive intake by humans resulting in a food intoxication, which gives rise to symptoms such as heat palpitations (Park et al., 2019). For histamine, the European Food Safety Authority (EFSA) panel has suggested fish containing less than 50 mg/kg is safe for consumption (EFSA, 2011). Our previous study has shown that the BA contents found in grasshopper sub shrimp pastes are not completely within the safe levels for consumption (Sang et al., 2020a). From the perspective of flavor, aldehydes, ketones, alcohols, esters, pyrazines, acids, and sulfides have been identified in the paste by HS-SPME-GC-MS analysis during the fermentation (Lv et al., 2020), and some of these substances, e.g., heptanal and n-hexanal, may produce unpleasant odors (Varlet et al., 2006). Starter culture application is a common practice for quality assurance of fermented foods and it offers a promising solution for accelerating the ripening speed of the food, as well as standardizing the quality and reducing safety hazards associated with fermented foods (Jeong et al., 2016).

The main microorganisms involved in fermentation, mainly lactic acid bacteria (LAB), are generally considered safe for consumption by humans. *Tetragenococcus muriaticus* is a member of LAB, which can tolerate the high salt concentration in the fermented foods. In many fermented products, *T. muriaticus* plays a crucial role in the production of nutrients and flavors (Jeyaram et al., 2008; Chen et al., 2012; Chuea-nongthon et al., 2017). Some studies have shown that in order to prevent the accumulation of BAs, selected strains of *Tetragenococcus* incapable of producing amines are often used as starter cultures for fermented food (Jeong et al., 2017; Wakinaka et al., 2019). In recent years, it has become a common practice to use bacteria with histamine-degrading enzyme to degrade the histamine in fermented food (Zaman et al., 2014; Kim et al., 2019). Nevertheless, bacterial growth and enzyme activities can be easily affected in some food by low pH, high temperature or high salinity. This limitation should be eliminated to ensure the efficacy of histamine-degrading bacteria in the food containing a high salt content, such as shrimp paste and fish sauce (Zaman et al., 2014; Kim et al., 2019). Only a few available reports have focused on bacteria that exhibit biogenic amine-degrading activity under such extreme environments. Meanwhile, an important prerequisite for starter cultures is the continuous presence of the relevant bacteria throughout the fermentation (Jeong and Lee, 2015). Recently, we have identified *T. muriaticus* as the predominant species present in grasshopper sub shrimp paste during the fermentation period (Sang et al., 2020b), demonstrating its potential as a starter culture for the production of this product.

In this study, we used high-throughput sequencing to analyze the prevalence of *T. muriaticus* in grasshopper sub shrimp paste samples collected from seven typical regions in China. We then determined the safety of these *T. muriaticus* strains by assessing their BA production, antibiotic susceptibility, hemolytic activity, and biofilm formation according to the EFSA guidelines for the safe use of microorganisms as food/feed materials. The salt tolerance, protease and lipase activities, acid production and amine oxidase of these *T. muriaticus* strains were also characterized to select the best possible starter culture. We also analyzed whether *T. muriaticus* could improve the flavor and BA- reduction in grasshopper sub shrimp paste.

## Experimental Methods

### Collection of Grasshopper Sub Shrimp Paste Samples

Using a previously described method (Sang et al., 2020a), a total of 63 final products were collected from 21 commercial grasshopper sub shrimp paste factories (three from each city) in seven typical regions around the Bohai Sea (Figure 1A).

### Analysis of Bacterial Diversity by High-Throughput Sequencing and Isolation of *T. muriaticus*

Bacterial diversity in the shrimp paste samples was analyzed by high-throughput sequencing, the accession number was SRP254601. *T. muriaticus* strains were isolated using a previously described method (Sang et al., 2020a). Briefly, the shrimp paste (10 g) from different regions were separately homogenized in 90 mL of distilled water inside a sterile stomacher bag and pummeled for a minute. The homogenate was serially diluted with distilled water and 100 μL of each dilution was plated on de Man-Rogosa-Sharpe (MRS) agar plates. All plates were incubated at 37°C for 2–7 d. Colonies with milky white and small round dot morphologies as many as possible were chosen for further analysis. To identify the bacteria, the *16S rRNA* sequence of each pure isolate was amplified by PCR, and the products were sequenced by BGI (Shenzhen, China).

### Determination of BAs Concentration by HPLC

The concentrations of BAs in the grasshopper sub shrimp paste samples and bacterial cultures were determined by HPLC as described in our previous study (Sang et al., 2020b).

### Determination of Minimum Inhibitory Concentrations (MICs)

MIC experiments were carried out in 96-well microplates. Ten antibiotics (ampicillin, chloramphenicol, ciprofloxacin, erythromycin, gentamicin, tetracycline, kanamycin, streptomycin, clindamycin, and spectinmycin) were selected among those frequently used to test the antibiotic susceptibility of LAB and five antibiotics (ofloxacin, sulfadiazine, furacillin, furazolidone, and sulfamethazine) were selected because of their frequent use in the culturing of marine animals. A 2-fold serial dilution was prepared for each antibiotic in the respective solvents (the solvents of ampicillin, gentamicin, kanamycin, streptomycin, and spectinmycin were deionized water; the solvents of chloramphenicol, erythromycin and tetracycline were ethanol; the solvent of ofloxacin was 0.85% sodium chloride solution; the solvent of ciprofloxacin was acetonitrile; the solvent of ofloxacin was 0.1M HCl; the solvent of furazolidone was chloroform; the solvents of sulfadiazine, furacillin and sulfamethazine were dimethyl sulfoxide) and the final antibiotic concentrations were between 0.5 and 512 mg/L. *T. muriaticus* strains were cultured in MRS broth containing 6% NaCl until they reached 10^8^ CFU/mL. The final inoculum density in each well was 5 × 10^5^ CFU/mL. The plates were then incubated at 36°C for 20 h under static conditions. The MIC for each antibiotic was recorded as the lowest concentration at which no turbidity was observed in the wells. Resistance to a particular antibiotic was defined as the point at which the MIC value for a tested antibiotic was higher than its recommended breakpoint value as defined by the European Committee on Antimicrobial Susceptibility Testing (EUCAST)^1^ in 2015 and 2020. Five independent experiments were conducted.

For the genus *Tetragenococcus*, the breakpoint values of the antibiotics have not been defined by the EFSA. Thus we referred to the breakpoint values provided by the EUCAST for *Enterococcus* in 2015 and 2020. Microbiological breakpoints are generally set by studying the distribution of the MICs of the chosen antimicrobial agents in a bacterial population belonging to a single taxonomical unit (species or genus). Strains with MICs higher than the breakpoints are considered resistant (EFSA, 2008).

### Hemolytic Activity Test

MRS agar supplemented with 5% (v/v) sheep blood (Solarbio, Beijing, China) was used for β-hemolytic activity tests. A loopful of bacteria was inoculated to blood plates and made the signs of the cross, then β-hemolytic activity was determined by cold shock at 4°C for 24 h after incubation at 37°C for 48 h (Jeong et al., 2017). Hemolytic activity was determined by the formation of clear lytic zones around the colonies on each blood-containing MRS. *Virgibacillus halodenitrificans* was used as a positive control. Five independent experiments were conducted.

### Biofilm Formation on 96-Well-Plate

Biofilm formation was measured using the microplate assay as described by Zhu et al. (2019). *Staphylococcus aureus* ATCC6538 was used as a positive control for the biofilm formation analysis. Five independent experiments were conducted.

### Determination of Salt Tolerance, Protease and Lipase Activities, and Acid Production

The salt tolerance of the isolated *T. muriaticus* strains deficient in histamine production was determined by examining their growth on MRS agar supplemented with NaCl up to 24% (w/v). Protease activity was determined using MRS agar containing 2% (w/v) skim milk, while lipase activity was tested using tributyrin agar (Solarbio) containing 1% (v/v) tributyrin. Each substrate-supplemented agar was aseptic drilled in advance, then bacterial solution cultured on MRS broth were transferred separately corresponding plates and incubated at 37°C for 48 h. The relative size of the clearing zone around the colony was used as an indicator of enzyme activity. Each culture (OD_600 *nm*_ = 0.4) was inoculated into NaCl supplemented medium and incubated at 37°C for 48 h, acid production was determined with a pH meter. The effect of NaCl on their protease and lipase activities, and acid production was determined by adding NaCl to the corresponding medium at a concentration of up to 24% (w/v).

### Amine Oxidase Experiment

Amine oxidase activity was measured using the method described by Ma et al. (2014). Briefly, a loopful of bacteria was dipped onto a piece of white clean filter paper. A drop of 1% dimethylphenylenediamine hydrochloride solution was added to the filter paper at the same spot. After that, a drop of 1% α-naphthol ethanol solution was then added, and any bacterial sample that gave rise to a blue color product within half a minute was regarded as positive with respect to the presence of amine oxidase activity.

### Evaluation of the Flavor in Grasshopper Sub Shrimp Paste Added by *T.muriaticus* Using an Electronic Nose

To evaluate the effect of *T. muriaticus* on grasshopper sub shrimp paste, each culture (OD_600 *nm*_ = 0.4) was inoculated into a separate sample of 50 g shrimp paste using an inoculum size of 4% (v/w). All samples were incubated for 10 days. The flavor of the samples was measured by a PEN3 portable E-nose (Win Muster Airsense Analytics Inc., Schwerin, Germany), which contains a detector unit composed of an array of 10 different metal oxide semiconductors (MOS)-type chemical sensors. The specific operation was carried out according to a described procedure (Xu L. et al., 2016).

### Histamine Degradation Ability Determination of *T. muriaticus* by HPLC

The ability of *T. muriaticus* to degrade histamine was determined according to a previously described procedure (Li et al., 2019), but with some modifications. Briefly, the cells were cultured in MRS broth containing 6% NaCl and collected by centrifugation at 6,000 × g for 5 min. After washing with 0.05 mol/L phosphate buffer (pH = 7), the cell pellet was diluted in phosphate buffer to an OD_600 *nm*_ of 0.4 containing 500 mg/L of histamine, cultured at 37°C 150 rpm for 48 h to detect the residual histamine in the suspension. The phosphate buffer without cell was used as a control. Three independent experiments were conducted.

### Reducing of BAs Formation in Grasshopper Sub Shrimp Paste Samples by *T. muriaticus*

To evaluate the ability of *T. muriaticus* to control the production of BAs present in grasshopper sub shrimp paste, the cells (OD_600 *nm*_ = 0.4) were inoculated into 50 g of shrimp paste using an inoculum size of 4% (v/w). The samples were incubated for 10 days, and the BA content was measured by HPLC. For negative control, an equivalent volume of sterile water was added to the paste instead of bacterial culture. Three independent experiments were conducted.

### Bioinformatics and Statistical Analysis

For the data of high-throughput sequencing, raw fastq files were quality-filtered by Trimmomatic and merged by FLASH. Operational taxonomic units (OTUs) were clustered with 97% similarity cutoff using UPARSE(version 7.1)^2^ with a novel “greedy” algorithm that performed chimera filtering and OTU clustering simultaneously. The taxonomy of each *16S rRNA* gene sequence was analyzed by RDP Classifier algorithm^3^ against the Silva 128/16s_bacteria database, using a confidence threshold of 70%. Spearman correlation heatmap diagram was performed with a software package of pheatmap in R language.

All statistical analyses were performed using the SPSS statistical program (Version on 22, IBM Co., Somers, NY, United States). The results were subjected to a one-way ANOVA analysis, with *p* < 0.05 considered as statistically significant. Data were expressed as mean ± standard deviation.

## Results

### Spearman Correlation Heatmap Analysis of Bacterial Communities and Histamine in Grasshopper Sub Shrimp Paste

Based on the *16S rRNA* gene sequence, *T. muriaticus* was found to be the predominant species in all the grasshopper sub shrimp paste samples and the abundance was particularly high in the Rongcheng, Panjin and Dalian samples (Figure 1B), representing up to 61.57, 49.84, and 26.09%, respectively.

**FIGURE 2:**
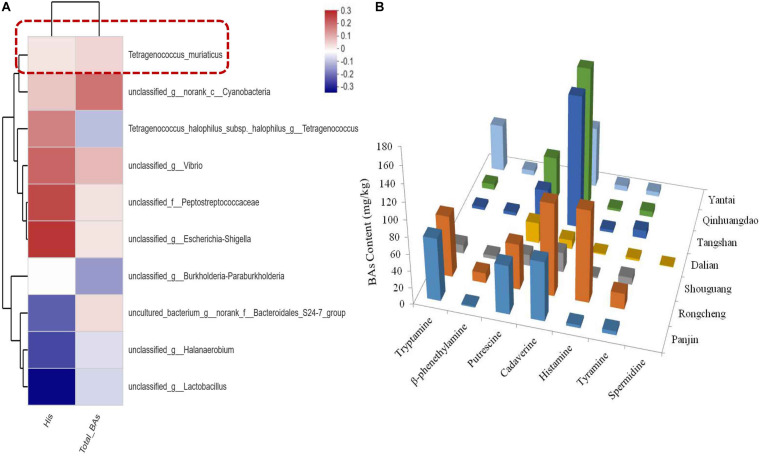
Correlation between bacterial community and histamine in grasshopper sub shrimp paste. **(A)** Heatmap dendrogram showing the correlation between *T. muriaticus* and BAs; **(B)** Contents of BAs in grasshopper sub shrimp paste samples.

Histamine was found in all the grasshopper sub shrimp paste samples (Figure 2B), and the total BA content varied among the grasshopper sub shrimp paste samples. The Rongcheng samples had the highest total BA content as well as the highest histamine concentrations, whereas the Dalian samples had the lowest total BA content.

The relationship between the abundance of individual species within the bacterial community (The top 10 in total abundance at species level) and BA content was evaluated by the spearman correlation heatmap analysis. Most of the species were found to exhibit a certain degree of correlation with BA contents (Figure 2A). *T. muriaticus* was weakly correlated with histamine (*R* = 0.00909, *p* = 0.96879) and total BA contents (*R* = 0.02857, *p* = 0.60769).

### Biogenic Amines Production by *T. muriaticus*

**FIGURE 3:**
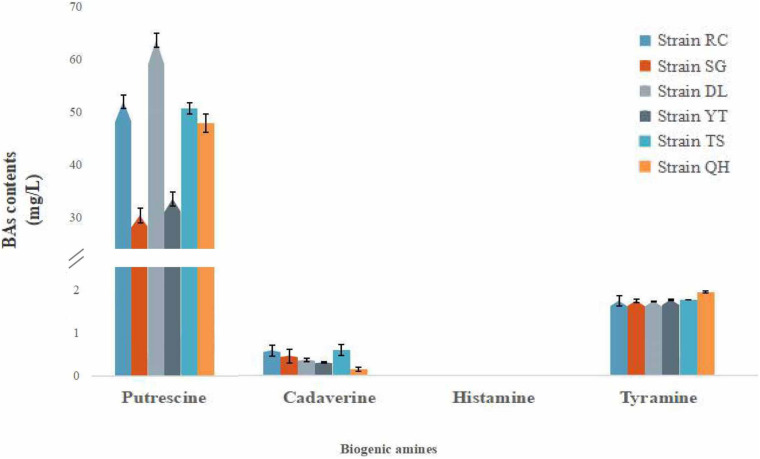
Biogenic amine production by *T. muriaticus* stains in MRS broth supplemented with precursors.

*16S rRNA* sequence analysis identified a total of 31 *T. muriaticus* strains from the shrimp paste samples of seven regions. Twenty-five of these strains were positive with respect to histamine production, and the average levels of putrescine, cadaverine, histamine and tyramine produced were 329.73, 184.51, 1.49, and 4.99 mg/L, respectively (Supplementary Table 1). The other six strains (RC, SG, DL, YT, TS and QH) did not produce histamine and the average levels of putrescine, cadaverine, and tyramine produced were 46.36, 0.41, and 1.82 mg/kg, respectively (Figure 3). These strains were isolated from Rongcheng, Shouguang, Dalian, Yantai, Tangshan and Qinhuangdao of China, and were used for subsequent experiments.

### Antibiotic Susceptibility, Hemolytic Activity, and Biofilm Formation

The six non-histamine producing *T. muriaticus* strains were each tested for susceptibility to a range of antibiotics, and the corresponding MIC values for each antibiotic are summarized in Table 1. The genus *Enterococcus* is phylogenetically most closely related to the genus *Tetragenococcus* based on taxonomic classification (Michael and Wilhelm, 1997; Jeong et al., 2017). Based on the values for *Enterococcus*, all the strains were sensitive to chloramphenicol, ciprofloxacin, gentamicin, ofloxacin and tetracycline. Strain RC showed higher resistance to ampicillin, whereas strain YT showed higher resistance to erythromycin, while strain DL and YT showed higher resistance to streptomycin. Their MIC values were all 2-fold higher than the breakpoints. Since clindamycin had no breakpoints and kanamycin, furacillin, furazolidone, spectinmycin, sulfadiazine, and sulfamethazine had no relevant standard in the breakpoint table of 2020 for the genus *Enterococcus*, we had to resort to the data of the MIC test to deduce the breakpoints for clindamycin, kanamycin, furacillin, furazolidone, spectinmycin, sulfadiazine and sulfamethazine, which were 4, 2, 16, 1, 4, 1, 4 mg/L, respectively. Generally speaking, the six *T. muriaticus* strains exhibited relatively weak resistance to the tested antibiotics.

**TABLE 1:** MIC distribution of fifteen antibiotics for *T. muriaticus* strains isolated from grasshopper sub shrimp paste.

**Antibiotic**	**Strains with MIC (mg/L)**												**Breakpoint (mg/L)^a^**
	**0.5**	**1**	**2**	**4**	**8**	**16**	**32**	**64**	**128**	**256**	**512**	**1,024**	
Ampicillin		TS	SG, QH	DL, YT	RC								4^a^
Chloramphenicol			DL, YT, TS, QH	RC, SG									32^b^
Ciprofloxacin			All										4^a^
Clindamycin			RC, DL, YT, TS, QH	SG									–
Erythromycin			RC, SG, TS, QH	DL	YT								4^b^
Gentamicin	SG, TS	RC, DL, YT, QH											128^a^
Ofloxacin		All											4^a^
Streptomycin										TS	RC, SG, QH	DL, YT	512^a^
Tetracycline		TS, QH	SG, DL	RC, YT									4^b^
Furacillin				RC, SG, TS	DL, QH	YT							
Furazolidone	SG, TS, QH	RC, DL, YT											
Kanamycin	QH	SG, DL, TS	RC, YT										
Spectinmycin	SG, TS	YT, QH	DL	RC									
Sulfadiazine	SG, DL, YT	RC, TS, QH											
Sulfamethazine				All									

*^a^The breakpoint values for Enterococcus provided by the European Committee on Antimicrobial Susceptibility testing (http://www.eucast.org/mic_distributions_and_ecoffs/) in 2020. ^*b*^The breakpoint values for Enterococcus provided by the European Committee on Antimicrobial Susceptibility testing (http://www.eucast.org/mic_distributions_and_ecoffs/) in 2015. –, have no breakpoints. Blank, there is no relevant standard in the breakpoint table of 2020.*

None of the strains exhibited β- hemolytic activity (data not shown) or produced detectable biofilm (Supplementary Table 2).

### Salt Tolerance, Protease and Lipase Activities, Acid Production and Amine Oxidase Experiment

All of the 6 *T. muriaticus* strains with non-histamine production grew on MRS agar containing 21% NaCl, but three could not maintain growth when the NaCl concentration in the medium was 24% or higher (Table 2). Most of the *T. muriaticus* isolates could produce acid in the presence of 18% NaCl, with maximum acid production occurring at 8% NaCl. The protease and lipase activities of *T. muriaticus* were strain-specific and both activities decreased as the NaCl concentration increased. Strain RC, SG, DL, YT, and TS maintained their protease activity at 10% NaCl and strain SG exhibited lipase activity at 4% NaCl. All six strains showed positive amine oxidase activity.

**TABLE 2:** Properties of the six *T. muriaticus* strains and the effect of NaCl on activity levels.

**Strains**	**Salt tolerance**			**Protease activities**			**Lipase activities**			**Acid production**			**Amine oxidase**
	**18%**	**21%**	**24%**	**6%**	**8%**	**10%**	**2%**	**3%**	**4%**	**1%**	**8%**	**18%**	
RC	+	w	−	+ ++	++	+	+	+	−	+	++	+	+
SG	+	+	w	+ ++	++	+	+	+	+	+	++	+	+
DL	+	+	−	+ ++	++	+	+	+	−	+	++	+	+
YT	+	+	w	+ ++	++	+	+	+	−	+	++	w	+
TS	+	w	−	+ ++	+	+	+	+	−	+	++	+	+
QH	+	+	w	+ ++	+	−	+	+	−	+	++	−	+

+, *positive activity; w, weak activity; −, *negative activity.**

### Evaluation of Histamine Degradation Properties by *T. muriaticus*

**FIGURE 4:**
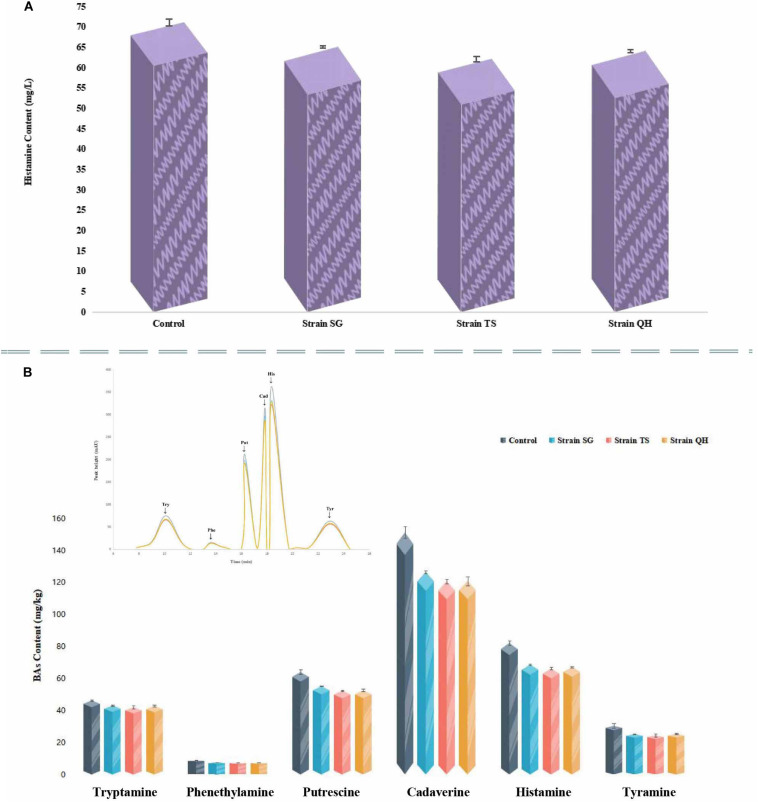
Evaluation of histamine degradation by *T. muriaticus* **(A)** and the effect of *T. muriaticus* on histamine accumulation in grasshopper sub shrimp paste **(B)**.

Strains RC, DL, and YT exhibited resistance to ampicillin, erythromycin and streptomycin were eliminated from the histamine degradation test. The ability to degrade histamine was investigated for the other three *T. muriaticus* strains (SG, TS, QH). After incubation for 48 h, all were positive with respect to histamine degradation. The histamine-degradation rates achieved by SG, TS, QH were 8.44, 12.60, and 9.88%, respectively (Figure 4A).

### Effects of *T. muriaticus* Strains on Histamine Accumulation and Flavor in Grasshopper Sub Shrimp Paste Samples

Strains SG, TS, and QH were each inoculated into separate shrimp paste and incubated for 10 days to determine their effects on histamine content (Figure 4B). After the incubation period, the histamine contents were 68.68 ± 0.56, 66.15 ± 0.80, and 67.21 ± 0.32 mg/kg, respectively, for the shrimp pastes incubated with SC, TS and QH, while the histamine content in the control shrimp paste was 82.23 ± 1.34 mg/kg. The concentration of histamine in the shrimp pastes was found to decrease significantly, with an average decrease of 18.10%. At the same time, the concentrations of tryptamine, β-phenylethylamine, putrescine, cadaverine, and tyramine were also decreased (the content in the control shrimp paste were 46.35 ± 0.54, 8.90 ± 0.11, 64.03 ± 1.33, 151.47 ± 3.54, 30.53 ± 1.31 mg/kg, respectively), with their residual concentrations being 42.66 ± 0.56, 7.40 ± 0.09, 53.39 ± 1.53, 122.70 ± 3.37, and 25.11 ± 0.51 mg/kg, respectively (Figure 4B).

**FIGURE 5:**
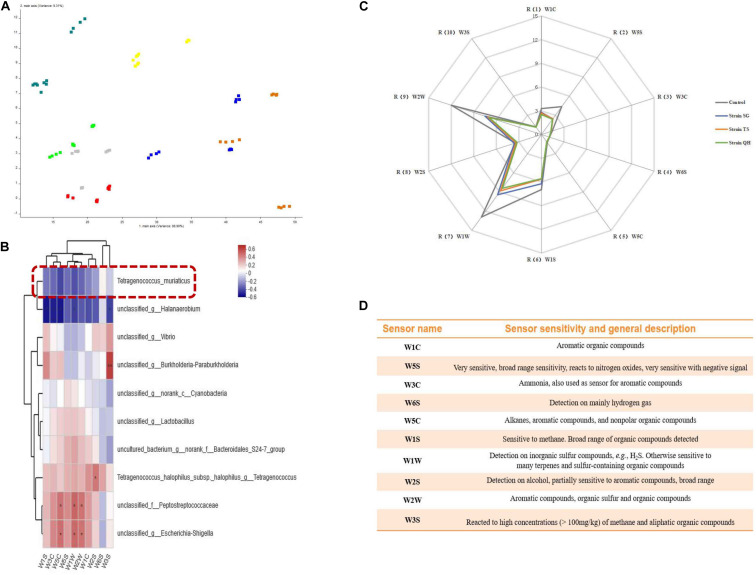
Evaluation of the flavor of grasshopper sub shrimp paste containing *T. muriaticus* by an electronic nose **(A)** PCA analysis of grasshopper sub shrimp paste samples; **(B)** Correlation between bacterial community and flavor compounds; **(C)** The changes of the flavor by adding *T. muriaticus* to shrimp paste samples; **(D)** Performance of each sensor.

The flavors in the shrimp pastes were determined by principal component analysis (PCA). By reducing the objects of the data into a few components without the loss of much information, PCA can be employed to interpret the data based on their differences and similarities for better visualization of all the information (Fan et al., 2017). In the models depicting the shrimp paste from different regions, the data points of the samples were scattered, and the contribution rates of PC1 and PC2 were 88.90 and 9.31%, respectively, giving a total contribution rate of 98.21% (Figure 5A). This suggested that the flavors were different for the different shrimp pastes, which could be distinguished by the electronic nose. The relationship between the abundance of individual species within the bacterial community (The top 10 in total abundance at the species level) and the response values of 10 sensors were evaluated by the spearman correlation heatmap analysis (Figure 5B). *T. muriaticus* was negatively correlated with the response values of 10 sensors (-0.42834 < *R* < 0.07472, 0.05271 < *p* < 0.74752). To verify the role of *T. muriaticus* in the flavor of grasshopper sub shrimp paste, strain SG, TS, and QH were separately inoculated into shrimp pastes and fermented for 10 days. The response values of the sensors W1W, W2W, W1S, W5S, W2S, and W1C were relatively high (Figure 5C), indicating that these six sensors were the most important factors that could discriminate the different shrimp paste samples. The characteristic flavor compounds were inorganic sulfide, organic sulfur, methyl, nitrogen oxides, alcohol and aromatic organic compounds (Figure 5D). The response values of the sensors underwent some changes following the addition of *T. muriaticus* strains to shrimp paste, suggesting that the flavor the shrimp pastes was improved, and this was likely caused by the presence of *T. muriaticus* strains (Figure 5C).

## Discussion

Grasshopper sub shrimp paste is a Chinese traditional fermented salted food that is prepared through spontaneous fermentation of the shrimps carried out by various microorganisms that are present in the raw materials or solar salts. The uncontrolled nature of the fermentation process makes it difficult to produce traditional fermented salty food commercially because of the issues associated with standardization. In addition, during the fermentation, the generation of BAs or toxins by the growth of undesirable microorganisms also prevents the commercialization of Chinese traditional fermented salty food (Gu et al., 2018). *Tetragenococcus* have been suggested as a suitable starter culture for the production of high quality standardized fermented salty food because they are frequently the dominant strains in fermented salty food (Gu et al., 2018). Recently, we have identified *T. muriaticus* as the stable dominant species found throughout the entire fermentation, and therefore, we believe that *T. muriaticus* could be the key bacterial group in grasshopper sub shrimp paste (Sang et al., 2020b). Furthermore, *16S rRNA* gene sequencing data revealed *T. muriaticus* as the prevailing species present in all the shrimp paste samples collected from the typical regions in China that produce grasshopper sub shrimp pastes (Figure 1). Its dominance and widespread presence may highlight its potential as a starter culture for the production of this product.

BAs have been reported in many fermented foods. Excessive amounts of BAs in food are undesirable because they can result in severe toxicological effects in humans, such as hypertension, headache, diarrhea, and localized inflammation and thus evaluating the BA content is particularly valuable since this could give some indication of food safety. In this study, seven common BAs were detected in sixty-three grasshopper sub shrimp paste samples, and the BA contents in the different shrimp paste samples showed relatively large variations. Although there are no regulations governing the permissible BA content in most foods, the US FDA has recommended an upper limit of 50 mg/kg for histamine in the edible portion of fish (Yang et al., 2014) and the EFSA panel considers fish containing histamine at less than 50 mg/kg to be safe for consumption (EFSA, 2011). According to the Chinese Standard GB2733 (2015), the maximum limit of histamine in fish such as mackerel permitted by law is 200–400 mg/kg in China. Based on the most stringent limits, it is worth noting that the concentration of histamine in some samples was found to exceed 50 mg/kg, indicating that there might be a safety risk. The relationships between a bacterial community and BA was evaluated by the spearman correlation heatmap analysis. *T. muriaticus* was found to weakly correlated with histamine and total BA contents (Figure 2), suggesting that *T. muriaticus* may contribute to reduce BA formation in shrimp paste.

It is beneficial to control the BA content of the fermented food by using low BA-producing strains as the starter cultures (Li et al., 2018). We isolated six *T. muriaticus* strains (RC, SG, DL, YT, TS, and QH) from grasshopper sub shrimp paste samples taken from different regions around the Bohai Sea. These strains were found to produce a low level of BAs but could not produce histamine, and therefore, they were subsequently subjected to safety assessment.

Antibiotic resistance is one of the major safety issues for the selection of starter culture. All the six *T. muriaticus* strains that unable to produce histamine were susceptible to common antibiotics, which are frequently used to test the antibiotic susceptibility of LAB. However, three of the strains (RC, YT, and DL) were resistant to ampicillin, erythromycin or streptomycin. Spectinmycin, ofloxacin, sulfadiazine, furacillin, furazolidone, and sulfamethazine are common antibiotics for aquatic marine animals, although they have no breakpoint or relevant standard, and the MICs of the antibiotics that we determined were less than 16 mg/L. Generally speaking, these *T. muriaticus* strains were only slightly resistant to the antibiotics tested, especially SG, TS and QH, which might be considered as safe for food application, at least in terms of their antibiotic resistance. Safety assessment is a matter of evidence accumulation gained from the history of use, as well as by planned research. Currently, there is no established research-based guideline for assessing the safety of shrimp paste in terms of its starter culture.

Besides BA formation and antibiotic susceptibility, we considered the hemolytic activity of the candidate starter cultures as specified in the FAO/WHO guidelines (FAO/WHO, 2002) and biofilm formation (Jeong et al., 2016) for assessing the safety associated with the usage of probiotic strains. Microbial strains with β-hemolytic activity produce exotoxins such as streptolysin that cause complete lysis of erythrocytes through the degradation of sphingomyelins (Jeong and Lee, 2015). The absence of hemolytic activity might eliminate potential risks of hemolysis linked to the bacterial strains used in the starter culture. Another major factor that can present a safety issue for the shrimp paste is the presence of biofilm-forming bacteria. Biofilm is a bacterial self-protection growth pattern whereby the pathogenic bacteria and spoilage bacteria can adhere to the solid surfaces that can come into contact with food. These bacteria may then form biofilms, and the biofilms will allow the cells to become more resistant to cleaning treatments, and enable them to contaminate the food during subsequent processing (Zhu et al., 2019). Biofilms have been recognized as a frequent source of bacterial infections (Costerton et al., 1999). None of the *T. muriaticus* strains isolated from the shrimp pastes was able to form biofilm, suggesting that it might be safe to use them in the production shrimp pastes.

Starter cultures have not previously been used to initiate the production of shrimp paste, thus the properties required for a successful starter culture have not been investigated. Considering the concentration of NaCl used in grasshopper sub shrimp paste production, salt tolerance would appear to be an important requirement for the starter culture. The six *T. muriaticus* strains were found to exhibit enough salt tolerance and acid-forming activity to contribute to the ripening of grasshopper sub shrimp paste through fermentation. Considering the rich protein contents of grasshopper sub shrimp and the rich shrimp oil of grasshopper sub shrimp paste products, the protease and lipase activities of the starter culture can help to improve the sensory qualities of the paste. Some differences in fermentation performance were observed among the different strains, although they all exhibited good salt tolerance, acid production, protease and lipase activities. This suggested that these bacterial strains could be used as starter culture to improve the flavor and quality of fermented products, such as shrimp paste.

Prevention of contamination, sanitary practices, and utilization of starter cultures are necessary to reduce BA production by microbes, which in turn could control the accumulation of BA during fermented food production (Park et al., 2019). Aquatic marine animals and their fermented products are rich in free amino acids, which make them susceptible to the activity of bacterial decarboxylase. Thus, these products might contain high levels of BAs, especially histamine. Taken together, the strains SG, TS and QH had higher safety index and better fermentation performance. Furthermore, they also possessed a high histamine degrading ability (Figure 4A). Thus, based on these properties, these three strains might be beneficial candidates for controlling the production of BAs in shrimp paste. Indeed, each of the strain produced much lower level of total BA compared with the control strain when inoculated into shrimp paste (the concentrations of tryptamine, β-phenylethylamine, putrescine, cadaverine, and tyramine in the shrimp pastes were found to decrease significantly, with an average decrease of 7.97, 16.77, 16.61, 19.00, and 17.75%, respectively). The histamine level in the shrimp paste inoculated with these strains also decreased by an average of 18.10%. This may be due to the interaction among multiple microbial species in a complex fermentation system (Sang et al., 2020b). The obvious reduction in BA level in the shrimp paste following the addition of *T. muriaticus* strains suggested that these bacterial strains could be used as potential candidates to control the BA level in fermented food. Other studies have shown that the salinity of the fermented food might be responsible for the different BA degradation rates in fish sauce and soy sauce, whereby high salinity would hinder the degradation of the BAs to some extent (Xu Y. et al., 2016). We speculated that a higher BA reduction rate might be obtained by inoculating these strains into fermented foods with lower salinity.

Shrimp pastes have unique textures, flavors and aromas mainly due to proteolysis and lipid degradation, the flavor is important for the evaluation of the product quality (Fan et al., 2017; Lv et al., 2020). However, like most of the traditional fermented foods, the quality of shrimp paste largely depends on the experience and technique of the maker. Given the accuracy of the results, the complexity of measurements and the cost of detection, E-nose is a better choice for identifying shrimp paste. According to the literature, dimethyl disulfide, dimethyl tetrasulfide, dimethyl trisulfide, 2, 3, 5-trimethyl-6-ethyl pyrazine, ethyl-2, 5-dimethyl-pyrazine, phenol, and indole were the typical volatile compounds contributing to the flavor of shrimp paste (Fan et al., 2017; Lv et al., 2020). Similar results were obtained in our study, with nitrogen and sulfur compounds being the main volatile flavor compounds in shrimp paste (Figure 5C). Shrimp pastes also have unpleasant flavor substances. The threshold value of alcohol is usually high, which has an aroma, plant flavor, rancidity and earthy mildew flavor whereas the threshold of aldehydes is generally low, which is an important flavor volatile in shrimp. On the other hand, the threshold value of ketones is much higher than that of aldehydes, which may enhance the flavor of shrimp paste (Luan et al., 2020). Heptanal has a strong, unpleasant, rough and pungent smell of grease while n-hexanal has oil and grass gas, and rancid and disgusting smell at high concentration (Luan et al., 2020). Similarly, propanoic acid, butanoic acid, furans, and 2-hydroxy-3-pentanone exhibit unpleasant odors, such as pungent and rancid odors (Fan et al., 2017). The spearman correlation heatmap revealed a negative correlation between *T. muriaticus* and ten flavor compounds (Figure 5B), demonstrating that *T. muriaticus* may play a role in improving the flavor of the shrimp paste during fermentation. The flavor of the paste samples containing *T. muriaticus* was weakened as a whole (Figure 5C). It could be inferred that the addition of *T. muriaticus* to the fermentation mixture could improve the flavor of grasshopper sub shrimp paste to make it milder and more acceptable to the consumers.

## Conclusion

This study is the first to assess the safety issue associated with *T. muriaticus* isolated from the shrimp pastes. Based on *16S rRNA* gene sequencing data, *Tetragenococcus muriaticus* was the predominant species in grasshopper sub shrimp pastes obtained from seven typical regions around the Bohai region of China. Six strains of *T. muriaticus* with low BA production and no histamine production exhibited sufficient salt tolerance, acid-formation, protease and lipase activities were isolated from these shrimp pastes. Furthermore, *T. muriaticus* was able to reduce BAs formation and improve the flavor of the shrimp paste to a great extent. Therefore, the isolated *T. muriaticus* strains could be used as potential candidates to control the production of BAs, especially histamine in fermented foods. Further study is required to investigate the environmental factors of fermentation along with other histamine-degrading bacteria that can influence the degradation of histamine in the shrimp paste, so that optimal controlling of histamine can be obtained.

## Data Availability Statement

The datasets presented in this study can be found in online repositories. The names of the repository/repositories and accession number(s) can be found in the article/Supplementary Material.

## Author Contributions

HHo and XS designed this study. XS conducted the experiments. XM and YZ performed the data analyses. HHo contributed reagents and materials. XS, HHa, JB, and GZ drafted and revised the manuscript. All authors read and approved the final version of this manuscript.

## Conflict of Interest

The authors declare that the research was conducted in the absence of any commercial or financial relationships that could be construed as a potential conflict of interest.

## Footnotes

1 http://mic.eucast.org

2 http://drive5.com/uparse/

3 http://rdp.cme.msu.edu/
