# Supplementary material for: Assessment of the Distribution and Safety of Tetragenococcus muriaticus for Potential Application in the Preparation of Chinese Grasshopper Sub Shrimp Paste
Source: Front Microbiol. 2021 Jan 28;12:628838. doi: 10.3389/fmicb.2021.628838 (PMC7876237; doi:10.3389/fmicb.2021.628838)
Supplement: Supplementary file 1 [file Data_Sheet_1.doc]

Supplementary Material

**Supplementary Table 1.** Biogenic amines production by *T. muriaticus* stains

| No. | Regions | mg/L | | | |
| --- | --- | --- | --- | --- | --- |
|  |  | Putrescine | Cadaverine | Histamine | Tyramine |
| 1 | Panjin | 380.03±45.11 | 119.86±14.10 | 1.15±0.05 | 4.82±0.13 |
| 2 | Panjin | 363.48±7.14 | 132.49±32.42 | 1.16±0.53 | 2.27±0.51 |
| 3 | Panjin | 335.32±6.68 | 146.12±9.02 | 1.62±0.24 | 1.91±0.06 |
| 4 | Panjin | 323.95±3.19 | 144.47±4.46 | 1.16±0.02 | 2.52±0.77 |
| 5 | Panjin | 354.49±3.34 | 106.89±13.67 | 2.00±0.79 | 6.95±1.71 |
| 6 | Panjin | 334.51±8.21 | 125.87±15.76 | 1.22±0.35 | 2.93±0.76 |
| 7 | Panjin | 348.08±7.49 | 98.02±7.36 | 1.74±0.81 | 4.97±0.60 |
| 8 | Panjin | 338.16±17.94 | 88.57±4.76 | 1.46±0.06 | 10.46±1.17 |
| 9 | Panjin | 326.73±3.35 | 94.28±3.55 | 0.80±0.31 | 4.16±1.45 |
| 10 | Panjin | 310.49±8.25 | 104.35±15.07 | 1.25±0.26 | 7.41±2.12 |
| 11 | Panjin | 337.56±11.87 | 36.64±5.12 | 1.26±0.22 | 2.00±0.28 |
| 12 | Panjin | 88.89±11.06 | 132.81±14.11 | 2.34±0.46 | 0.00±0.00 |
| 13 | Panjin | 158.41±6.97 | 4.90±0.37 | 1.51±0.12 | 1.74±0.36 |

Continued

| 14 | Panjin | 82.76±13.70 | 1.62±0.21 | 2.31±0.77 | 1.58±0.13 |
| --- | --- | --- | --- | --- | --- |
| 15 | Rongcheng | 364.97±13.99 | 132.60±12.33 | 1.77±0.54 | 2.75±1.80 |
| 16 | Rongcheng | 352.06±10.59 | 143.07±8.98 | 1.84±0.43 | 1.78±0.20 |
| 17 | Rongcheng | 304.00±13.37 | 109.07±5.50 | 1.43±0.05 | 2.05±0.50 |
| 18 | Rongcheng | 223.81±22.19 | 6.60±1.99 | 1.62±0.27 | 1.79±0.19 |
| 19 (QC) | Rongcheng | 51.99±1.28 | 0.59±0.55 | 0.00±0.00 | 1.77±0.13 |
| 20 | Shouguang | 347.05±10.81 | 78.68±11.84 | 1.70±0.56 | 1.78±0.07 |
| 21 (SG) | Shouguang | 30.37±1.40 | 0.46±0.16 | 0.00±0.00 | 1.78±0.04 |
| 22 | Dalian | 337.76±10.32 | 130.07±24.62 | 1.65±0.19 | 1.87±0.08 |
| 23 | Dalian | 143.27±21.69 | 5.35±0.05 | 1.46±0.10 | 2.98±1.82 |
| 24 (DL) | Dalian | 63.66±1.35 | 0.37±0.04 | 0.00±0.00 | 1.77±0.01 |
| 25 (YT) | Yantai | 33.52±1.30 | 0.30±0.02 | 0.00±0.00 | 1.80±0.02 |
| 26 | Tangshan | 370.00±8.21 | 126.94±0.44 | 1.34±0.18 | 2.56±0.93 |
| 27 | Tangshan | 342.52±8.07 | 79.33±7.69 | 1.44±0.05 | 1.68±0.05 |
| 28 | Tangshan | 343.64±6.04 | 64.70±8.54 | 1.36±0.08 | 1.76±0.14 |
| 29 | Tangshan | 338.81±5.52 | 65.28±8.12 | 1.31±0.08 | 1.73±0.12 |
| 30 (TS) | Tangshan | 50.74±1.08 | 0.61±0.57 | 0.00±0.00 | 1.81±0.00 |
| 31 (QH) | Qinhuangdao | 47.88±1.74 | 0.15±0.02 | 0.00±0.00 | 1.99±0.02 |

**Supplementary Table 2.** The results of biofilm formation (n=5)

| Strains | OD_600nm_ | OD_590nm_ |
| --- | --- | --- |
| Blank | -0.545±0.229 | -0.029±0.005 |
| *Staphylococcus aureus* ATCC6538 | 0.431±0.101 | 0.300±0.038 |
| RC | 0.360±0.108 | -0.015±0.017 |
| SG | 0.420±0.142 | -0.012±0.009 |
| DL | 0.356±0.090 | -0.015±0.015 |
| YT | 0.372±0.059 | -0.015±0.011 |
| TS | 0.302±0.113 | -0.017±0.013 |
| QH | 0.330±0.073 | -0.022±0.007 |
